# Impact of Benzo(a)pyrene and Pyrene Exposure on Activating Autophagy and Correlation with Endoplasmic Reticulum Stress in Human Astrocytes

**DOI:** 10.3390/ijms26041748

**Published:** 2025-02-18

**Authors:** Tanapan Siangcham, Pornpun Vivithanaporn, Kanyaluck Jantakee, Jittiporn Ruangtong, Nattaya Thongsepee, Pongsakorn Martviset, Pathanin Chantree, Phornphan Sornchuer, Kant Sangpairoj

**Affiliations:** 1 Faculty of Allied Health Sciences, Burapha University, Chonburi 20131, Thailand; tanapan@go.buu.ac.th; 2 Chakri Naruebodindra Medical Institute, Faculty of Medicine Ramathibodi Hospital, Mahidol University, Samut Prakan 10540, Thailand; pornpun.viv@mahidol.edu; 3 Thammasat University Research Unit in Nutraceuticals and Food Safety, Faculty of Medicine, Thammasat University, Pathum Thani 12120, Thailand; kanyaluckjan@gmail.com (K.J.); jittiporn.rt@gmail.com (J.R.); nattayat@tu.ac.th (N.T.); pong_m@tu.ac.th (P.M.); pathanin@tu.ac.th (P.C.); psmicro@tu.ac.th (P.S.); 4 Department of Preclinical Science, Faculty of Medicine, Thammasat University, Pathum Thani 12120, Thailand

**Keywords:** benzo(a)pyrene, pyrene, astrocyte, autophagy, ER stress, polycyclic aromatic hydrocarbons, neurotoxicity

## Abstract

Benzo(a)pyrene (B(a)P) and pyrene, the most prominent subtypes of polycyclic aromatic hydrocarbons (PAHs), contaminate environments as organic pollutants. They adversely affect body systems, including degeneration of the central nervous system. This study investigated the in vitro toxic effects of B(a)P and pyrene on proliferation, endoplasmic reticulum (ER) stress induction, and autophagy in human astrocytes using U-87 MG human astrocytoma cells as a model. Both B(a)P and pyrene were toxic to U-87 MG cells in a concentration-dependent manner. Astrocytic proliferation was interfered with, enhancing S-phase cell cycle arrest. B(a)P promoted the presence of autophagic vesicles and the expression of autophagic markers LC3, beclin-1, and p62, suggesting activated autophagy. B(a)P enhanced the expression of ER stress markers BiP, PERK, and IRE1. ER stress appeared to be correlated with autophagy induction, as demonstrated by experiments using chloroquine, an autophagy inhibitor. Pyrene enhanced the expression of autophagic markers and ER stress primarily via PERK activation, although autophagic vesicles were not observed. The study demonstrates that B(a)P enhances ER stress-mediated autophagy more evidently than pyrene and affected toxicity to astrocytes. These results provide a basis for understanding the toxic effects of the main PAH substances affecting astrocytes.

## 1. Introduction

Polycyclic aromatic hydrocarbons (PAHs) are ubiquitous environmental pollutants which originate from petrogenic, pyrogenic, and biogenic sources [1]. Exposure to PAH can be detrimental to the nervous system as neurotoxic agents, as presented by several reports. A systematic review revealed global research on PAH neurotoxicity, with a focus on neurodegeneration, cholinergic function, neurodevelopmental toxicity, behavioral change, and oxidative stress [2]. Prenatal PAH exposure by accumulation in umbilical cords at high concentrations may be associated with an increased risk for fetal neural tube defects [3]. PAH mixtures in particulate matter, containing primarily benzo(a)pyrene and pyrene, are the main contaminants of air pollution.

Benzo(a)pyrene (B(a)P) is the most characterized member of the PAH family; its contamination in found in several environments including air, soil, water, and foods. B(a)P exhibits carcinogenic effects in various human organs and in animal models. Additionally, B(a)P exposure is associated with mutagenesis, genotoxicity, immunotoxicity, and developmental toxicity. Previous studies have demonstrated the promotion of migration, invasion, and angiogenic abilities of B(a)P in several cancers [4,5]. Due to its liposolubility, B(a)P accumulates in lipid-containing tissues, including the brain [6,7]. B(a)P can be used as a reliable indicator of PAH occurrence or contamination in foods [8].

Pyrene, composed of four fused benzene rings, is present in high concentrations as a component of almost all PAH mixtures. Pyrene has been shown to promote hepatotoxicity and renal dysfunction, as evidenced by increased liver metabolizing enzymes, depletion of hepatic GSH level, and elevated inflammatory and renal damage markers [9,10]. Exposure to low-level environmental concentrations of pyrene has been reported to cause cardiotoxicity in zebrafish embryos [11]. Pyrene has also been found to exhibit hepatoxicity in human primary hepatocytes by activating mouse constitutive androstane receptor (CAR) and increasing CYP2B6 mRNA expression, independently of aryl hydrocarbon receptor (AhR) response [12]. Metabolomic analysis in hepatocytes following low-dose pyrene exposure revealed notable alterations in hepatic metabolism, particularly in glycerophospholipid metabolism [13]. Co-exposures of pyrene or its derivative with UV irradiation induce toxicity, oxidative stress, and DNA damage in human keratinocytes [14,15].

Astrocytes are the main cellular subtypes in the central nervous system (CNS). They are essential for maintaining key functions such as homeostasis, metabolic function, antioxidation and inflammatory response in CNS [16]. Damage to astrocytes disrupts their homeostasis, leading to neurotoxicity, neurodegeneration, and death. Astrocytes are highly sensitive to traffic-related air pollutants and, responding to systemic inflammation, act as the first sensors for brain-penetrating pollutant particles [17]. However, the toxicity of PAHs, including the effects of B(a)P and pyrene on astrocyte damage, has not been investigated.

Autophagy is an essential, evolutionarily conserved catabolic cellular process for the turnover and recycling of constituents of unwanted or damaged cells. Astrocyte autophagy is induced in response to several harmful factors including metabolic restriction, oxygen deprivation, neuroinflammation, and neurodegeneration to promote protection from harmful stressors and to promote survival not only of themselves but also of other neural cells [18,19,20]. A previous study strongly suggested that basal autophagy is essential for neuronal survival [21]. However, excessive autophagic processes impair cellular morphology and function, potentially resulting in autophagic death. B(a)P exposure caused injury and induction of autophagic and pyroptotic death of human hepatic cells [22], but no previous finding has been reported regarding the autophagic effects of pyrene. Exposure to PAH substances might target central nervous tissues, impairing astrocyte proliferation and function. This study revealed toxic effects of B(a)P or pyrene on proliferation and induction of autophagy, correlating with the endoplasmic reticulum (ER) stress response.

## 2. Results

### 2.1. Prediction of Blood–Brain Barrier Permeability Effect of B(a)P and Pyrene

**Figure 1:**
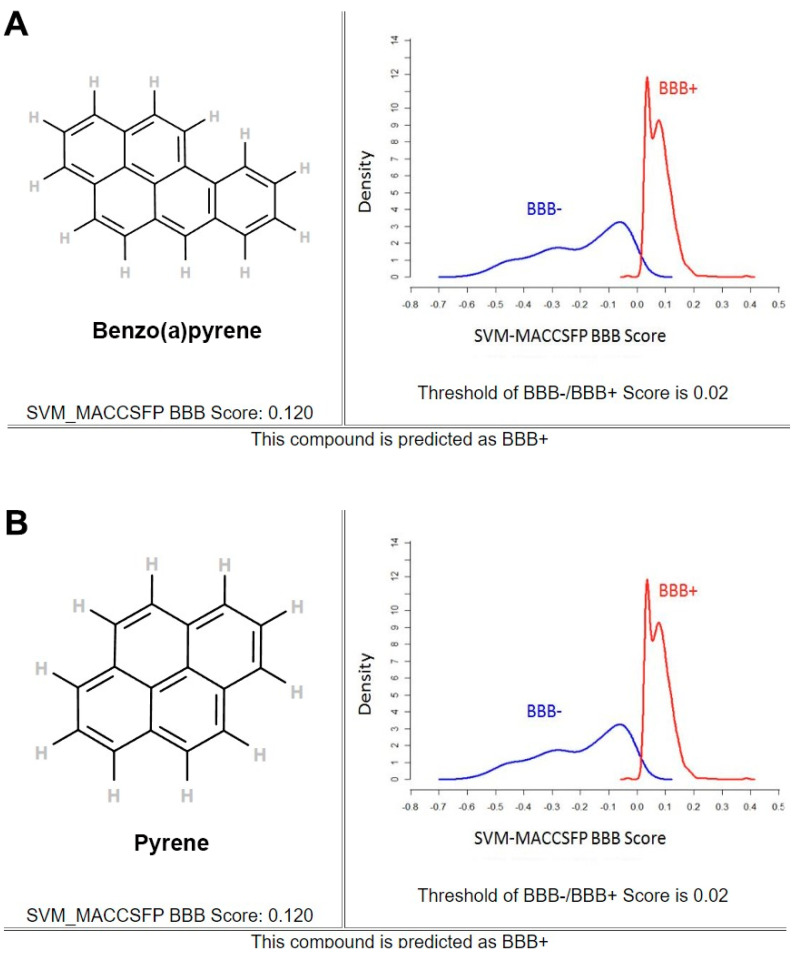
The predicted BBB permeability of B(a)P (**A**) and pyrene (**B**) obtained from Online BBB Predictor (https://www.cbligand.org/BBB/, accessed on 1 June 2024).

To investigate whether B(a)P and pyrene can penetrate the BBB and affect CNS cells, in silico predictions of blood–brain barrier (BBB) permeability for both substances were conducted based on their chemical properties using an online tool which was available at https://www.cbligand.org/BBB/, accessed on 1 June 2024. This online tool employs two different algorithms, AdaBoost and Support Vector Machine (SVM), combining with four different fingerprints, namely MACCS, OpenBabel, Molprint 2D, and PubChem, employed to classify whether a compound was BBB-permeable (BBB+) or BBB-impermeable (BBB−) [23]. Predicted permeability scores higher than the threshold score of 0.02 assumed that a compound was the BBB+. Regarding polarity, B(a)P and pyrene have no polar surface area (0 A2), so they both potentially could pass through the BBB. For both B(a)P and pyrene, the online tool calculated a permeability score of 0.120 (Figure 1), indicating both substances can potentially permeate the BBB. This implied that B(a)P and pyrene could have biological consequences on CNS cells.

### 2.2. Cytotoxicity

**Figure 2:**
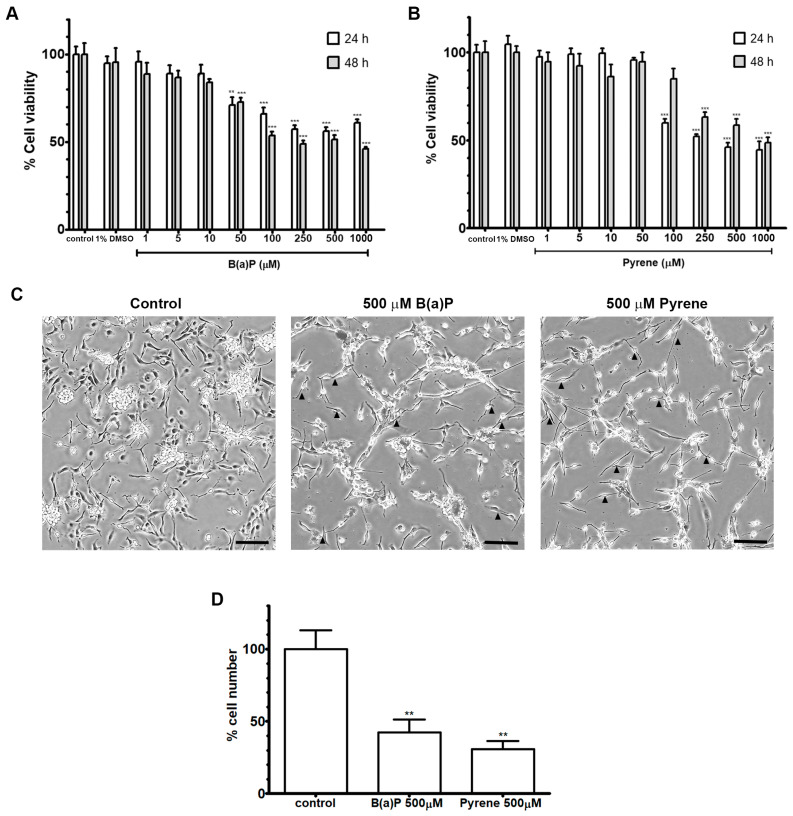
Viability of U-87 MG cells after exposure to B(a)P (**A**) and pyrene (**B**) for 24 and 48 h was measured by MTT assay. The percentage of viability in each concentration was compared with the untreated control (*n* = 5). ** *p* < 0.01, *** *p* < 0.001 (**C**) The representative photomicrograph of morphological change in U87-MG cells after exposure to B(a)P or pyrene at 500 μM for 48 h. Arrowheads indicate flattened cytoplasm and straight processes. (Scale bar = 50 µm). (**D**) The percentage of U-87 MG cell number after exposure to B(a)P and pyrene at 500 μM for 48 h compared with the untreated control (*n* = 3). ** *p* < 0.01.

The effect of B(a)P and pyrene on viability of U-87 MG human astrocyte cells was determined using an MTT assay following 48 h post-exposure. Both compounds affected the viability of U87-MG cells in a similar pattern. B(a)P-exposed cells at low concentrations (1 to 10 μM) have stably high viability. Upon exposure to 50 μM of B(a)P, viability was slightly decreased to about 50% of viability until high concentrations (1000 μM) (Figure 2A). Pyrene-exposed cells at concentrations from 1 to 100 μM also have stably high viability, then decrease to about 50% when exposed to 250–1000 μM (Figure 2B). Observation of morphological changes found that U-87 MG cells exposed to B(a)P or pyrene at 500 μM for 48 h enhanced a decrease in cell number and density with a flattened cytoplasm and straight processes (pointed as arrowheads) when compared with untreated control group which showed oval or pyramidal cytoplasm with most cone-shaped processes (Figure 2C,D). This indicated that B(a)P and pyrene exert moderate toxicity on astrocytes at the desired concentrations and time point.

### 2.3. Analysis of Cell Proliferation

**Figure 3:**
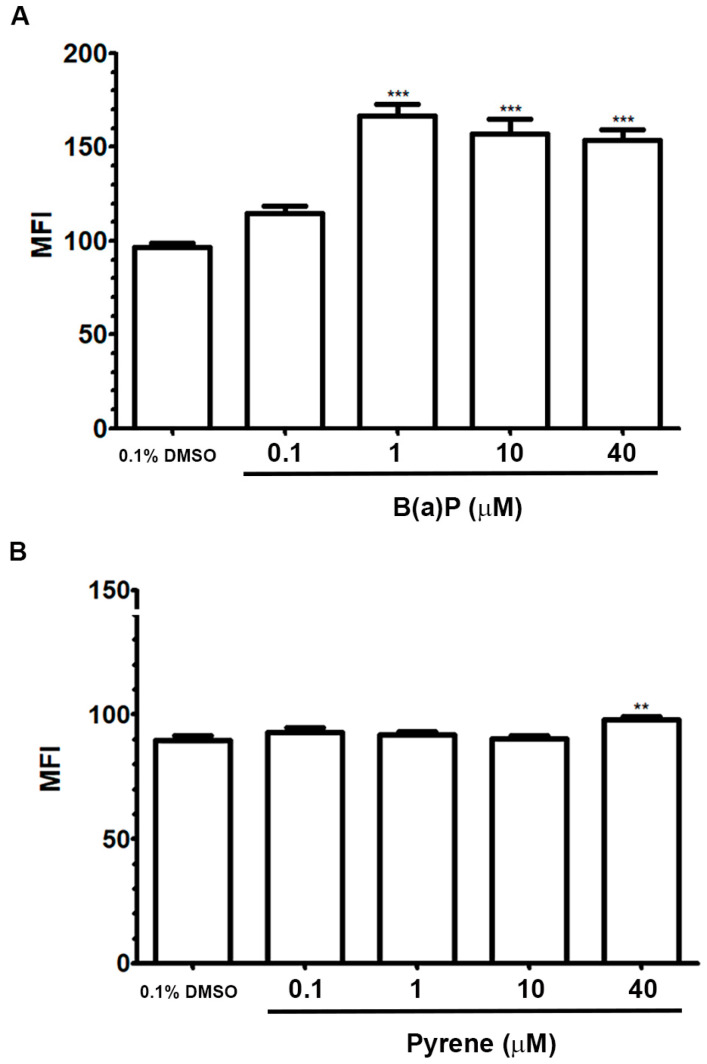
Effect on cell proliferation. The proliferation of U-87 MG cells was analyzed by CFSE assay. The mean fluorescence intensity (MFI) detected in cells following B(a)P (**A**), and pyrene (**B**) exposures for 96 h was compared with 0.1% DMSO-treated control (*n* = 3). ** *p* < 0.01, *** *p* < 0.001.

Effects of B(a)P or pyrene on proliferation of astrocytes were assessed using CFSE assays. In this test, mean fluorescence intensity (MFI) decreases as cells divide, indicating greater cell proliferation. After 96 h post-exposure, the MFI of B(a)P-exposed groups at 1, 10, and 40 μM had significantly increased compared to 0.1% DMSO-treated control group (Figure 3A). Pyrene exposure at 0.1–10 μM did not significantly change the MFI level compared to the control group, but it was slightly increased when exposed to 40 μM of pyrene (Figure 3B). This implies that B(a)P and pyrene have an anti-proliferative effect in astrocytes, with B(a)P exhibiting the greater effect.

### 2.4. Analysis of Cell Cycle

**Figure 4:**
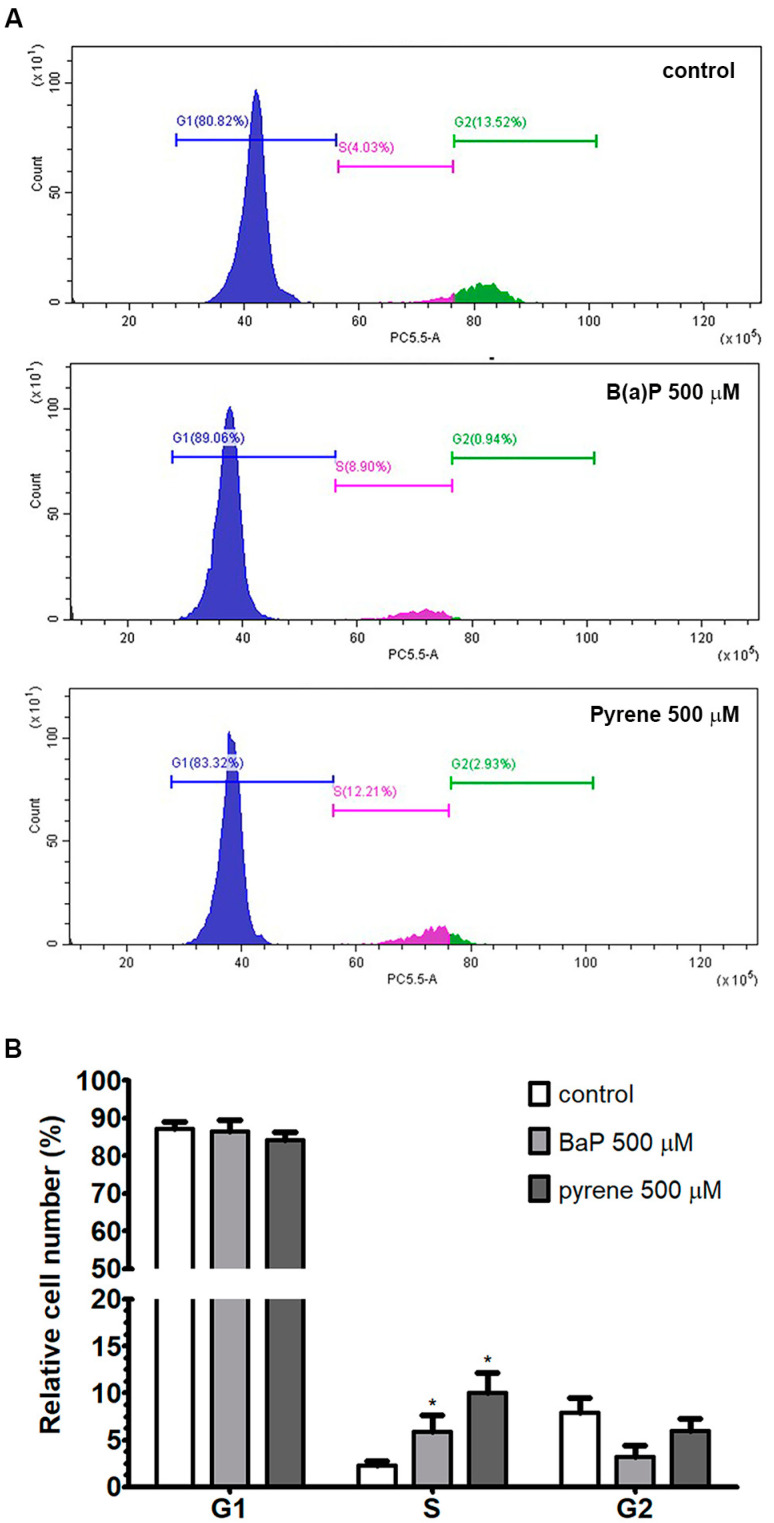
(**A**) Analysis of cell cycle. Change of cell cycle phases in U-87 MG cells after exposure to B(a)P and pyrene for 48 h was shown as the histograms of propidium iodide intensity. (**B**) The bar graph represents the percentage of cell number in each phase compared with 0.1% DMSO-treated control (*n* = 3). * *p* < 0.05.

Cell cycle progression in U87-MG cells following exposure to B(a)P and pyrene was detected by PI/RNase assay using flow cytometry. After 48 h incubation, B(a)P and pyrene exposures at 500 μM increased the percentage of U87-MG cell distribution at S phase, while decreasing the proportion of cells at G1 and G2-M phases (Figure 4). This result indicated that B(a)P and pyrene at 500 μM promoted cell cycle arrest at S phase in U87-MG cells.

### 2.5. Autophagic Flux

**Figure 5:**
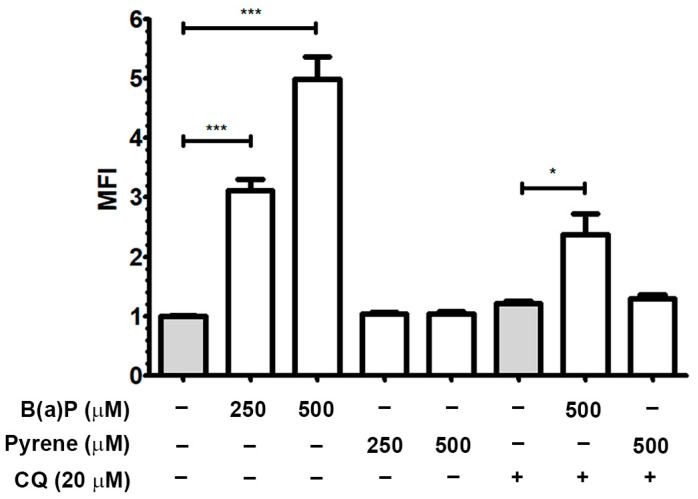
Effect on induction of cellular autophagy. Induction of autophagic flux on B(a)P and pyrene-treated U-87 MG cells after incubation for 48 h was detected by the CYTO-ID^Ⓡ^ autophagy assay staining. The bar graph represents the green fluorescence intensity detected in treated cells compared with 0.1% DMSO or CQ-treated controls (*n* = 3). * *p* < 0.05 and *** *p* < 0.001.

The effects of B(a)P or pyrene exposures on induction of cellular autophagy in U-87 MG cells were observed by the cyto-ID assay. At 48 h post-exposure, fluorescence signals indicating autophagic vesicles were strongly detected in the B(a)P-exposed group but less so in the pyrene group. The MFI of B(a)P-exposed groups at 250 and 500 μM was significantly increased compared to the untreated control. But the MFI of the pyrene-exposed groups remained unchanged compared to the control (Figure 5). To confirm autophagy induction, B(a)P at 500 μM was co-treated with the autophagy inhibitor CQ at 20 μM. B(a)P without CQ increased the MFI more than the CQ-treated group. There was insignificant change when pyrene was co-treated with CQ. This implies that B(a)P induces cellular autophagy in U-87 MG cells.

### 2.6. Expressions of the Autophagy Marker Proteins

**Figure 6:**
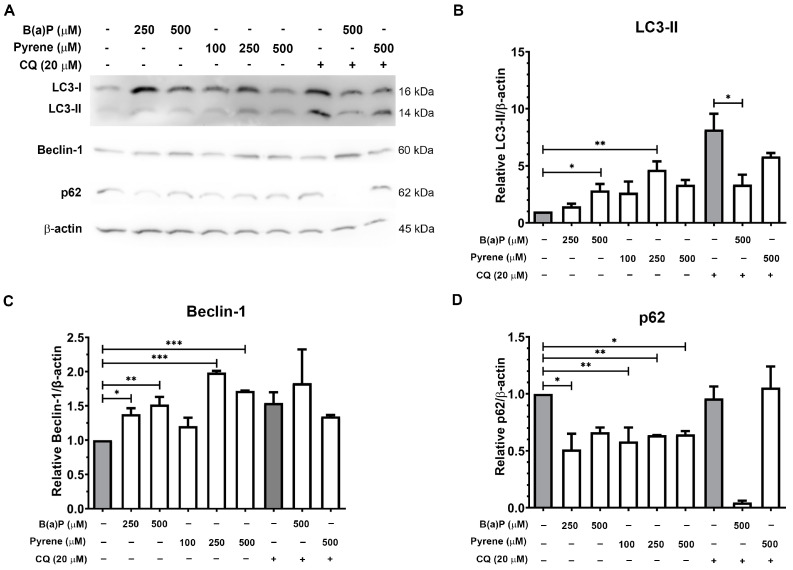
Analysis of autophagic protein expression level. (**A**) Immunoblot analysis of autophagic markers LC3, beclin-1, and p62 expression in U-87 MG cells after exposure to B(a)P and pyrene for 48 h compared with 0.1% DMSO- or CQ-treated control groups. (**B**–**D**) The relative expression levels of LC3-II, beclin-1, and p62 proteins were normalized to β-actin expression level and compared with DMSO or CQ control (*n* = 3). * *p* < 0.05, ** *p* < 0.01, *** *p* < 0.001.

Expression of autophagy markers following B(a)P or pyrene exposure was investigated by immunoblotting. At 48 h post-exposure, 250 and 500 μM of B(a)P had increased expression levels of LC3-II and beclin-1 and decreased expression of p62 (Figure 6). Co-treatment of 500 μM B(a)P or pyrene with 20 μM CQ decreased LC3-II expression level. B(a)P with CQ substantially decreased p62, but pyrene with CQ did not affect p62 expression. Beclin-1 expression was increased when co-treated with B(a)P and CQ but decreased slightly when co-treated with pyrene and CQ. These results indicate that B(a)P and pyrene are correlated with mediating the expression of autophagic marker proteins in U87-MG cells.

### 2.7. Expression of ER Stress-Related Proteins and Correlation with Autophagy

**Figure 7:**
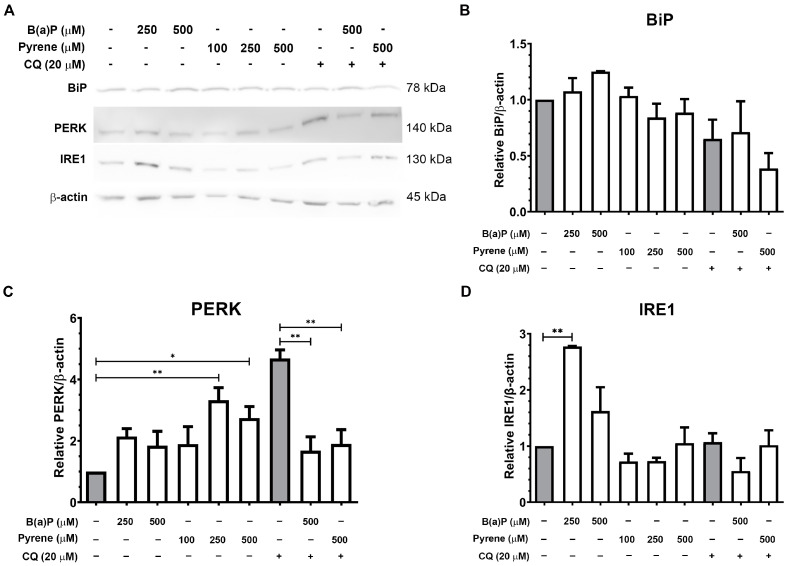
Analysis of ER stress marker protein expression level. (**A**) Immunoblot analysis of ER stress markers BiP, PERK, and IRE1 in U-87 MG cells after exposure to B(a)P and pyrene for 48 h compared with 0.1% DMSO- or CQ-treated control groups. (**B**–**D**) The relative expression levels of BiP, PERK, and IRE1 proteins were normalized to β-actin expression level and compared with DMSO or CQ control (*n* = 3). * *p* < 0.05, ** *p* < 0.01.

The expression of ER stress marker proteins BiP, PERK, and IRE1 in U87-MG cell following exposure to B(a)P or pyrene for 48 h was analyzed by immunoblotting. B(a)P-exposed cells at 250 and 500 μM showed increased expression of BiP, PERK, and IRE1. Pyrene exposure increased PERK expression but did not significantly change BiP and IRE1 expressions. To investigate whether ER stress response is related to autophagy in PAH-exposed astrocytes, the expression of ER stress markers in PAH-exposed cells co-treated with CQ was analyzed. Compared with the untreated control, CQ reduced BiP, and increased PERK expression levels. Co-treatment with CQ and B(a)P reduced PERK and IRE1 expression levels but minimally increased in BiP intensity compared with the CQ-treated group. CQ with pyrene reduced BiP and PERK expressions but left IRE1 unchanged compared with the CQ-treated group (Figure 7). This implies that B(a)P or pyrene exposures could mediate ER stress, which may correlate with autophagy induction as shown in B(a)P-exposed U-87 MG cells.

## 3. Discussion

PAHs, including B(a)P and pyrene, persistently disperse throughout the environment and contaminate food. Human exposure to PAHs poses a significant risk of toxicity to essential cells or tissues. Several studies have revealed multiple neurotoxic effects of B(a)P arising from impairing cellular differentiation to cognitive functions, which leads to enhanced neurodegeneration [24]. Since B(a)P and pyrene were found as PAH mixtures in the environment, the study aims to investigate the toxic effect of individual B(a)P or pyrene exposure to compare their toxicity and anti-proliferative effects.

Exposure with B(a)P or pyrene in human astrocytes causes moderate toxicity at moderate-to-high concentrations. Additionally, PAHs have an anti-proliferative effect on astrocytes. We found that B(a)P and pyrene reduce the division of U-87 MB cells into daughter cells by arresting their cell cycle progression in the S phase. The expression of cell cycle markers reflecting the arrest after PAH exposure, especially in chronic exposure conditions, would be investigated as a continuation of the study. Disruption of the cell cycle progression might be caused by a cellular stress response to cytotoxicity. A morphological change in astrocytes observed after PAH exposure may reflect astrocyte senescence, a functional change in the astrocyte from neurosupportive to neuroinflammatory, marked by enlargement with flattened cell morphology, growth arrest, and secretion of pro-inflammatory markers [25]. Further studies on the correlation of PAH exposure and astrocyte senescence are ongoing by our group.

Autophagy is an important cellular process that regulates cell activity, including that of central nervous cells. It may play a role in neurotoxicity induced by exposure to environmental pollutants [26]. Astrocytes may promote an autophagy process to clear disease-related or damaged proteins and maintain their normal function. However, excessive progression of autophagy may damage astrocytes, resulting in neurodegeneration [27]. We found that B(a)P exposure results in the accumulation of autophagosomes in U-87 MG cells, evidence of induction of cellular autophagy. Detection of autophagic markers after B(a)P exposure showed an increased expression of LC3-II and beclin-1, along with a decrease in p62 expression, indicating autophagy induction. The increased LC3-II can be a marker of either increased autophagy or the inhibition of autophagy due to the blockade of the autophagosome proteasomal clearance. The degradation of p62, which is usually incorporated into the autophagosome as a cargo receptor, is a marker for active autophagy [28]. The decreased p62 expression level following B(a)P exposure indicates the induction of autophagy. This effect was further confirmed by treatment with CQ. CQ can increase the pH of lysosomes, inhibiting fusion between lysosomes and autophagosomes and blocking autophagic flux [29]. As CQ blocks the autophagosome at the final step of autophagy, active LC3-II is not fully utilized, allowing accumulation of the protein. CQ and B(a)P-exposed cells promoted LC3-II utilization because of autophagy induction, resulting in decreased protein expression compared with the CQ-treated group. The inductive effect of B(a)P on autophagy is also revealed by reverse expression of beclin-1 and p62 in the co-treated CQ+B(a)P group compared with the CQ-treated group. Previous studies have shown that B(a)P exposure induced autophagic and pyroptotic death and arrested the cell cycle at the S phase by inhibiting the PI3K/Akt signaling pathway in HL-7702 human normal liver cells [22,30]. Mice treated orally with B(a)P at 75 mg/kg for 28 consecutive days upregulated autophagic markers LC3-II and beclin-1 with caspase-3 expressions in the brain, together with a marked increase in the malondialdehyde (MDA) level and decrease of glutathione (GSH) content in the brain [31]. Many studies observed neurotoxicity of environmental pollutants including lead, methylmercury, cadmium, and dioxin through mediating autophagy [26]. By pyrene exposure, autophagic markers LC3-II and beclin-1 were slightly upregulated with p62 expression downregulated but autophagic cells were not detected. This may be due to the autophagy-independent function of LC3 or beclin-1 that is activated to control other cellular functions, such as LC3-associated phagocytosis and cytokinesis [32]. A previous study revealed LC3-associated phagocytosis of photoreceptor outer segments in a p62-independent manner, which may be associated with Nrf2 activation and resulted in impaired autophagic degradation [33]. The correlation of autophagosome detection and autophagic protein expression may not equal causation, thus further mechanistic studies would be needed to confirm this conclusion. Autophagy activation also works in tandem with ER stress as the important cellular stress to maintain homeostasis [34].

ER stress is characterized by the chronic accumulation of aberrant proteins, which disturbs the protein folding capacity of the ER. Mild to moderate ER stress-induced unfolded protein response (UPR) signaling is related to a compensatory mechanism, whereas severe ER stress induced cell death through apoptosis activation and increased autophagic processes to remove damaged cells [35]. Accumulation of misfolded proteins and ER stress induction in CNS cells create a toxic milieu that contributes to neuronal dysfunction and inflammatory response from surrounding glia cells [36]. Autophagy is activated after severe ER stress induction in neuroblastoma cells for their survival, as observed by the presence of the autophagosome [37]. Induction of the UPR markers PERK, ATF6, and IRE1 are all related to activating the autophagy through multistep pathways [33]. ER stress and autophagy play critical roles in various CNS cells, including astrocytes, in both healthy and various neurodegenerative diseases [38]. This study found that the autophagy-induced effect of B(a)P in U-87 MG cells was correlated with increased expression of PERK, IRE1, and BiP as ER stress sensing markers. Pyrene upregulated only PERK expression, without significant changes in IRE1 and BiP. The inhibition of autophagy by CQ results in the accumulation of damaged proteins in the cytoplasm and induces ER stress [39]. Focusing on PERK and IRE1 as the main ER sensor, CQ increased their expression, which decreased after co-treatment with B(a)P or pyrene. There is a correlation between expression of beclin-1 and BiP in autophagy-induced cells as previously found [40]. Compared with CQ treatment, BiP increased, associated with increased beclin-1 when co-exposed with B(a)P but decreased by pyrene. These results imply that B(a)P promotes autophagy mediated by the response to ER stress through PERK, IRE1, and BiP pathways. Benzo(a)pyrene-7,8-diol-9,10-epoxide, a B(a)P derivative, decreased viability with activated ER stress response markers by increasing PERK and GRP78 expression and was associated with pyroptosis in human bronchial epithelial cells [41]. Pyrene may enhance ER stress, primarily via PERK activation, but may not be associated with autophagy. This may be due to the mild expression level of ER stress markers that are not sufficient to activate autophagy progression under these experimental conditions. The toxic effect of long-time pyrene exposure on enhancing autophagy or other types of cell death might be further elucidated. This study may contain some limitations. A single type of immortalized cell line was used in this study, which may not reflect the real situation of central nervous tissue interactions. This study was performed at a high concentration of both PAH substances to distinguish autophagic effects which are not obviously seen at lower concentrations. A further study on neuroinflammation and other cellular effects on astrocytes after exposure to low-to-moderate as well as chronic exposure of PAHs might also be further elucidated. In addition, a further study design using an in vitro co-culture incorporating CNS cells to investigate their interaction might be warranted.

## 4. Materials and Methods

### 4.1. Determination of BBB Permeability

Blood–brain barrier (BBB) permeation scores of 2D-structure of B(a)P (https://pubchem.ncbi.nlm.nih.gov/rest/pug/compound/CID/2336/record/SDF?record_type=2d&response_type=display, accessed on 1 June 2024) and pyrene (https://pubchem.ncbi.nlm.nih.gov/rest/pug/compound/CID/31423/record/SDF?record_type=2d&response_type=display, accessed on 1 June 2024) were derived using the BBB prediction server (https://www.cbligand.org/BBB/mainpage.php, accessed on 1 June 2024) [23].

### 4.2. Cell Culture and Treatment

U-87 MG human astrocytoma cell lines were obtained from American Type Culture Collection (ATCC, Rockville, MD, USA) and cultured in Minimal Essential Medium (MEM) supplemented with 10% FBS and a 1% penicillin/streptomycin cocktail in an incubator with an atmosphere of 5% CO2 at 37 °C. B(a)P and pyrene (Sigma, St. Louis, MO, USA) were prepared in DMSO and dissolved in DMEM prior to cell treatment. Autophagy inhibitor chloroquine (CQ) (Enzo Life Sciences Inc., Farmingdale, NY, USA) was dissolved in DMEM at the final concentration of 1 mM to evaluate correlation with autophagy.

### 4.3. Cytotoxicity Testing

U-87 MG cells cultured in 96-well plates at about 70% confluency were incubated for 24 h with increasing concentrations of B(a)P and pyrene diluted in serum-free MEM. The final concentration of DMSO was 0.25%. The cytotoxicity following B(a)P and pyrene treatments was determined by methyl thiazolyl tetrazolium (MTT) assays. MTT solution (Bio Basic, Markham, ON, Canada) was diluted in treated wells for a final concentration of 30 μg/mL, then incubated for 3 h. After that, DMSO was added to dissolve MTT formazan. The reaction was measured by a microplate reader (Varioskan Flash microplate reader, Thermo Fisher Scientific, Waltham, MA, USA) at the absorbance wavelengths of 562 and 630 nm. In addition, morphological change of the exposed cells was visualized using a phase-contrast microscope (Nikon, Tokyo, Japan) at 4× magnification.

### 4.4. Cell Proliferation Assay

U87-MG cells were labelled with 1 µM carboxyfluorescein succinimidyl ester (CFSE) dye (Sigma, USA) in dark at 37 °C for 10 min, the excess dye was washed, and they were seeded in a media containing 5% FBS on 6-well plates. Cells were treated with B(a)P or pyrene for 48 h, then retreated with the compound for another 48 h. After that, cells were trypsinized and resuspended in phosphate buffered saline (PBS). A fluorescence signal in living cells was detected using a Guava easyCyte flow cytometer (Merck Millipore, Bedford, MA, USA) at a minimum of 5000 events/sample.

### 4.5. Cell Cycle Analysis

U-87 MG cells were treated with 500 μM B(a)P or pyrene for 48 h. Treated cells were harvested with 0.1% trypsin-EDTA and fixed in methanol for 30 min. Cells were incubated with propidium iodide (PI)/RNase (BD Biosciences, San Jose, CA, USA) for 30 min. The fluorescence intensity of the PI-stained cells were measured using a DxFlex flow cytometer (Beckman Coulter, Brea, CA, USA) at a minimum of 10,000 events/sample.

### 4.6. Analysis of Cellular Autophagy

Following B(a)P and pyrene treatments, autophagy in U-87 MG cells was evaluated using the CYTO-ID^®^ Autophagy Detection Kit (Enzo Life Sciences Inc., USA) to specifically detect all autophagic vesicles in the cells. In brief, U-87 MG cells treated in 96-well plates for 24 h were incubated with green detection reagent in DMEM medium without phenol red for 30 min at room temperature. CQ (20 μM) was used as the negative control. Green fluorescence signals indicating the presence of autophagic vesicles were measured by a fluorescence microplate reader (Varioskan LUX multimode microplate reader, Thermo Fisher Scientific, USA) at excitation and emission wavelengths of 480 and 530 nm, respectively.

### 4.7. Immunoblotting

Whole proteins from treated cells were extracted using a radioimmunoprecipitation assay cell lysis buffer (Cell Signaling Technology, Inc., Danvers, MA, USA). Concentrations of protein lysates were determined using Bradford protein assays (Bio-Rad Laboratories Inc., Hercules, CA, USA). Equivalent amounts of proteins (20 μg each) were separated by 15% SDS-PAGE and transferred onto nitrocellulose membranes (Cytiva, Marlborough, MA, USA). The membranes were blocked in Tris-buffered saline and Tween-20 (TBST) containing 5% bovine serum albumin for 1 h. The samples were incubated with the primary antibodies, including rabbit anti-light chain 3 (LC3) (1:1000), rabbit anti-p62 (1:1000), rabbit anti-beclin1 (1:1000), rabbit anti-binding immunoglobulin-protein (BiP) (1:1000), rabbit anti-inositol-requiring enzyme 1 α (IRE1α) (1:1000), rabbit anti-protein kinase RNA-like endoplasmic reticulum kinase (PERK) (1:1000), and rabbit anti-β-actin (1:5000) (Cell Signaling Technology, Inc.) at 4 °C overnight. The membranes were washed with TBST and incubated with horseradish peroxidase-conjugated secondary antibodies (Cell Signaling Technology, Inc.) diluted in 0.01 M TBST at 1:5000 dilutions at room temperature for 1 h. The expression of targeted proteins was visualized using an enhanced chemiluminescence system (Thermo Fisher Scientific, Waltham, MA, USA). The relative expression level of target proteins was normalized to β-actin expression and compared with the control group.

### 4.8. Statistical Analysis

Data were expressed as mean ± standard deviation on at least triplication. Statistical variations of all experiments were analyzed with GraphPad Prism 9 statistical analysis software (GraphPad Software Inc., San Diego, CA, USA) using a one-way ANOVA test followed by Dunnett’s multiple comparisons test. *p* < 0.05, two-tailed, is considered significance.

## 5. Conclusions

The study demonstrates that B(a)P and pyrene, the main PAH substances in environmental pollutants, decrease proliferation, arrest the cell cycle at S phase, and mediate ER stress markers in U-87 MG human astrocytes. B(a)P may notably promote autophagy and be associated with mediation of the ER stress response. These results provide a basis for understanding the toxic effects of the main PAH substances affecting astrocytes.

## Data Availability

The raw data supporting the conclusions of this article will be made available by the corresponding authors without undue reservation.
